# S‐GMAS: Genome‐Wide Mediation Analysis With Brain Subcortical Shape Mediators

**DOI:** 10.1002/hbm.70297

**Published:** 2025-07-31

**Authors:** Shengxian Ding, Rongjie Liu, Anuj Srivastava, Richard S. Nowakowski, Li Shen, Paul M. Thompson, Heping Zhang, Chao Huang

**Affiliations:** ^1^ Department of Biostatistics Yale University New Haven Connecticut USA; ^2^ Department of Statistics University of Georgia Athens Georgia USA; ^3^ Department of Statistics Florida State University Tallahassee Florida USA; ^4^ Department of Biomedical Sciences Florida State University Tallahassee Florida USA; ^5^ Department of Biostatistics, Epidemiology and Informatics, Perelman School of Medicine University of Pennsylvania Philadelphia Pennsylvania USA; ^6^ Imaging Genetics Center, USC Mark and Mary Stevens Neuroimaging and Informatics Institute, Keck School of Medicine University of Southern California Los Angeles California USA; ^7^ Department of Epidemiology & Biostatistics University of Georgia Athens Georgia USA

**Keywords:** Alzheimer's disease, corpus callosum, elastic shape representation, genome‐wide mediation analysis

## Abstract

Mediation analysis is widely utilized in neuroscience to investigate the role of brain image phenotypes in the neurological pathways from genetic exposures to clinical outcomes. However, it is still difficult to conduct mediation analyses with whole genome‐wide exposures and brain subcortical shape mediators due to several challenges including (i) large‐scale genetic exposures, that is, millions of single‐nucleotide polymorphisms (SNPs); (ii) nonlinear Hilbert space for shape mediators; and (iii) statistical inference on the direct and indirect effects. To tackle these challenges, this paper proposes a genome‐wide mediation analysis framework with brain subcortical shape mediators. First, to address the issue caused by the high dimensionality in genetic exposures, a fast genome‐wide association analysis is conducted to discover potential genetic variants with significant genetic effects on the clinical outcome. Second, the square‐root velocity function representations are extracted from the brain subcortical shapes, which fall in an unconstrained linear Hilbert subspace. Third, to identify the underlying causal pathways from the detected SNPs to the clinical outcome implicitly through the shape mediators, we utilize a shape mediation analysis framework consisting of a shape‐on‐scalar model and a scalar‐on‐shape model. Furthermore, the bootstrap resampling approach is adopted to investigate both global and spatial significant mediation effects. Finally, our framework is applied to the corpus callosum shape data from the Alzheimer's Disease Neuroimaging Initiative.

## Graphical abstract

Using the ADNI Corpus Callosum (CC) shape data, we proposed a genome‐wide mediation analysis framework involving high‐dimensional genetic exposures and shape mediators and successfully identified distinct genetic‐to‐clinical outcome pathways mediated through the CC in Alzheimer's Disease, revealing varying spatial causal effect patterns for different clinical outcomes.

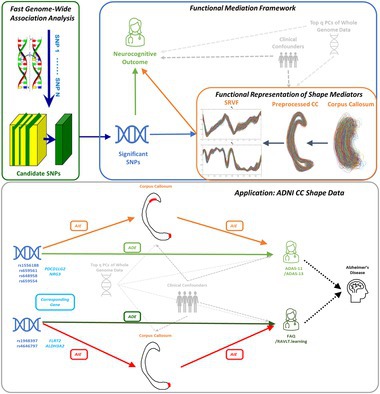

## 1 Introduction

In the past decade, with the rapid growth of large‐scale neuroimaging genetics studies, like the Alzheimer's Disease Neuroimaging Initiative (ADNI) study (Mueller et al. 2005), genome‐wide association studies (GWAS) with neuroimaging phenotypes represent an exciting and rapidly evolving area of research that integrates genetics and neuroimaging to better understand the biological basis of brain structure, function, and related disorders (Hibar et al. 2011; Huang et al. 2017; Nathoo et al. 2019; Shen and Thompson 2019; Zhao et al. 2021; Zhu et al. 2023). Unlike traditional GWAS, which focus on categorical disease traits or simple quantitative phenotypes, GWAS in neuroimaging genetics utilizes continuous and high‐dimensional (Huang, Nichols, et al. 2015) neuroimaging data as phenotypes, offering insights into intermediate traits or endophenotypes that lie between genetics and clinical manifestations (Huang et al. 2017). Recently, one of the most important scientific questions in neuroimaging genetics is whether and how genetic exposure affects mental health through the changes in brain microstructures, typically treated as a causal mediation analysis problem (Bi et al. 2017; Le and Stein 2019; Chen et al. 2022; Zhao and Li 2022; Zhou et al. 2024). Typically, structural equation models (SEMs) (VanderWeele 2015) are employed to investigate the causal direct effect of genetic exposures on neurocognitive outcomes and the indirect effect through neuroimaging mediators (Zhao and Li 2022; Bao et al. 2023; Dai and Zhang 2024; Zhou et al. 2024), where the neuroimaging phenotypes in the SEM can encompass univariate or multivariate variables (e.g., Region of Interest (ROI) based volumetric data; Zhao and Li 2022; Bao et al. 2023), high dimensional variables (e.g., connectome data; Dai and Zhang 2024), and functional representations (e.g., ROI surface data; Zhou et al. 2024).

Despite substantial scientific advancements in recent years aimed at improving mediation analysis in imaging genetics, conducting such analyses with whole‐genome‐wide exposures and brain subcortical shape mediators remains challenging due to several factors. First, most current studies in neuroimaging genetics focus on low‐dimensional genetic exposures, typically considering a few candidate genetic variants like single‐nucleotide polymorphisms (SNPs) (Zhao and Li 2022; Zhou et al. 2024). However, with millions of SNPs collected in neuroimaging genetics studies, it is crucial to identify relevant genetic exposures from thousands of variants and examine their effects on neurocognitive outcomes mediated by neuroimaging biomarkers. Second, although various neuroimaging mediators have been examined in existing mediation studies, limited research has explored scenarios involving complex structural mediators, such as the shapes of brain subcortical regions (Zhou et al. 2024). Subcortical shapes play a critical role in understanding the causes of various mental disorders. For instance, the shapes of the corpus callosum (CC) and hippocampus have attracted attention in Alzheimer's Disease (AD) research due to significant atrophy observed in AD patients compared to healthy controls (Huang et al. 2017; Zhou et al. 2024). While volumetric measures capture localized variations in tissue concentration (Whitwell 2009), shape‐based representations model the intrinsic geometric properties and spatial deformation patterns of anatomical structures. These shape features can reveal coordinated, global morphological alterations that may not be fully captured by voxel‐wise or surface morphometric measures, providing complementary and potentially more sensitive information for mediation analysis. In particular, subcortical shape is defined as the characteristic remaining after removing shape‐preserving transformations like rotations, translations, and scaling from vertex coordinate information on the subcortical surface (Small 1996; Srivastava and Klassen 2016). Consequently, shape representation spaces are nonlinear, high‐dimensional, and possess quotient space geometry (Huang, Styner, and Zhu 2015; Huang et al. 2021; Zhou et al. 2024), making conventional normality assumptions used in existing structural equation models (SEMs) unsuitable for subcortical shape data. Furthermore, for subcortical shape phenotypes, it is important to investigate both the overall indirect genetic effects on neurocognitive outcomes through shape mediators and the local indirect effects through alterations in subregions of the subcortical surface. Hence, developing statistical inference tools to assess both global and local indirect effects is of significant importance.

**FIGURE 1:**
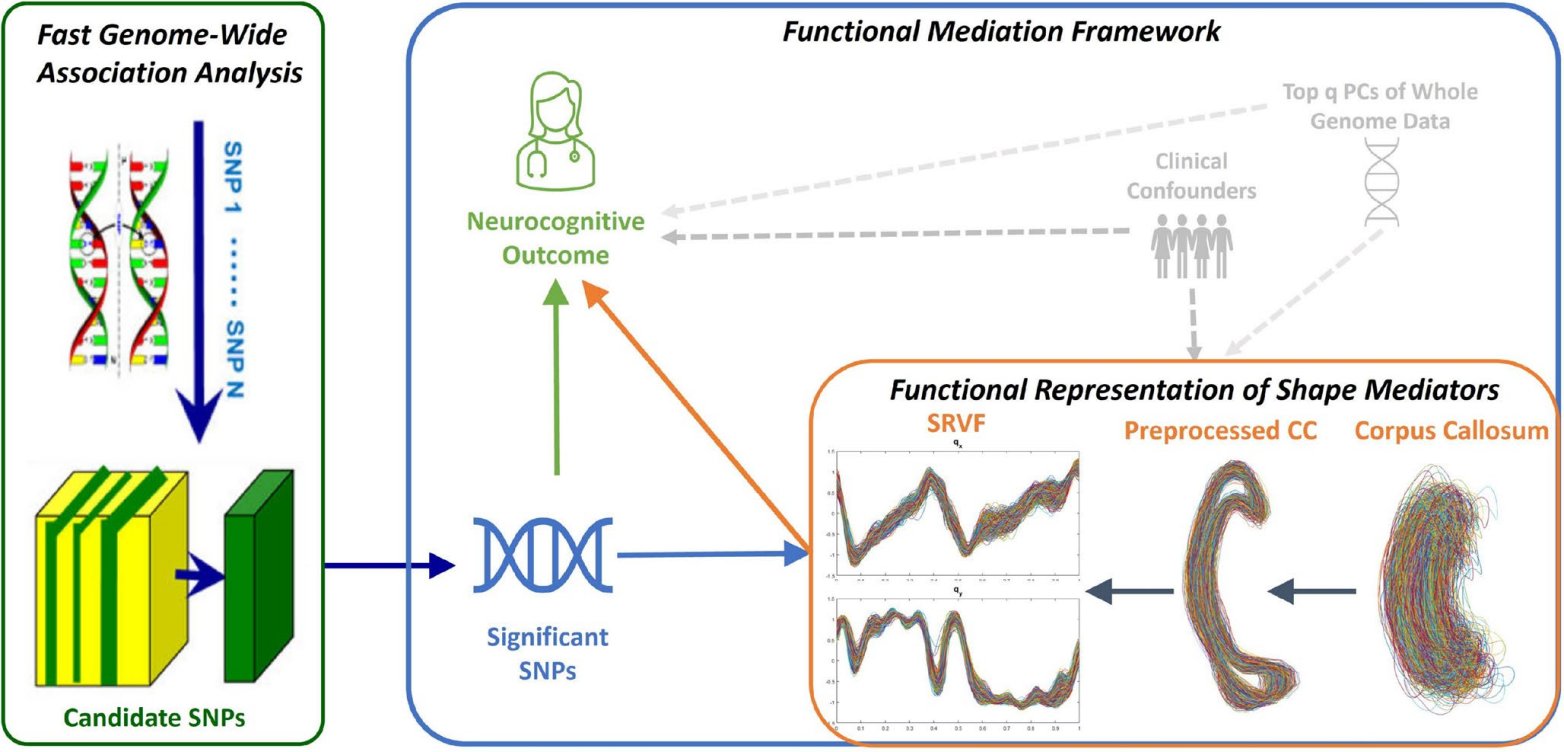
The S‐GMAS framework includes (i) a fast genome‐wide association analysis, (ii) functional representations of shape mediators, and (iii) a functional mediation framework.

To address all the challenges mentioned above, this paper aims to introduce a novel framework for Genome‐wide mediation analysis with shape mediators (S‐GMAS) to investigate the role of subcortical shape alterations in the neurological pathways from genetic exposures to neurocognitive outcomes. As shown in Figure 1, our imaging genetics mediation analysis framework, S‐GMAS, consists of three key components: first, to address the issue caused by the high dimensionality in genetic exposures, a fast genome‐wide association analysis (fastGWAS) proposed by van Rossum and Kruijer (2023) is conducted to discover potential genetic variants (i.e., SNPs) with significant genetic effects on the neurocognitive outcome; second, the shape information is extracted from the subcortical brain structure and represented as functions in linear Hilbert space; thirdly, by treating these shape representations as functional phenotypes, a functional mediation analysis model is established to identify the underlying causal pathways from the detected genetic exposures to the neurocognitive outcome implicitly through the subcortical shapes, including a shape‐on‐scalar regression model and a scalar‐on‐shape regression model. Furthermore, a bootstrap resampling approach is adopted to construct confidence intervals and simultaneous confidence bands (SCBs) for investigating both global and spatial significant mediation effects. Finally, our framework is applied to the ADNI CC shape data, and we successfully identify the mediation effect of a subset of candidate SNPs on Alzheimer's Disease, corresponding to causal genes of AD revealed in previous literature. By analyzing different neurocognitive outcomes of AD, we successfully detected one subregion (the posterior) of CC on outcomes designed for early AD detection and found two subregions (the anterior and posterior) of the CC on outcomes assessing severe cognitive impairments. Moreover, simulation studies through synthetic datasets are conducted to explore the estimation performance of the unknown functions and parameters and investigate the causal estimands. A sensitivity analysis is performed to examine the influence of unobserved confounders.

The paper is organized as follows. Section 2 introduces the ADNI study's large‐scale imaging and genetic dataset. Section 3 introduces our framework S‐GMAS and presents the estimation and inference procedures for the causal estimands. In Section 4, a comprehensive mediation analysis based on ADNI Corpus Callosum data is conducted, comprising the comparisons between (i) using the volumetric representations and shape representations as the mediators, and (ii) using the Polygenic Hazard Score (PHS) and genome‐wide single‐nucleotide polymorphisms (SNPs) as the genetic exposure. The conclusions are given in Section 5.

## 2 Materials

**FIGURE 2:**
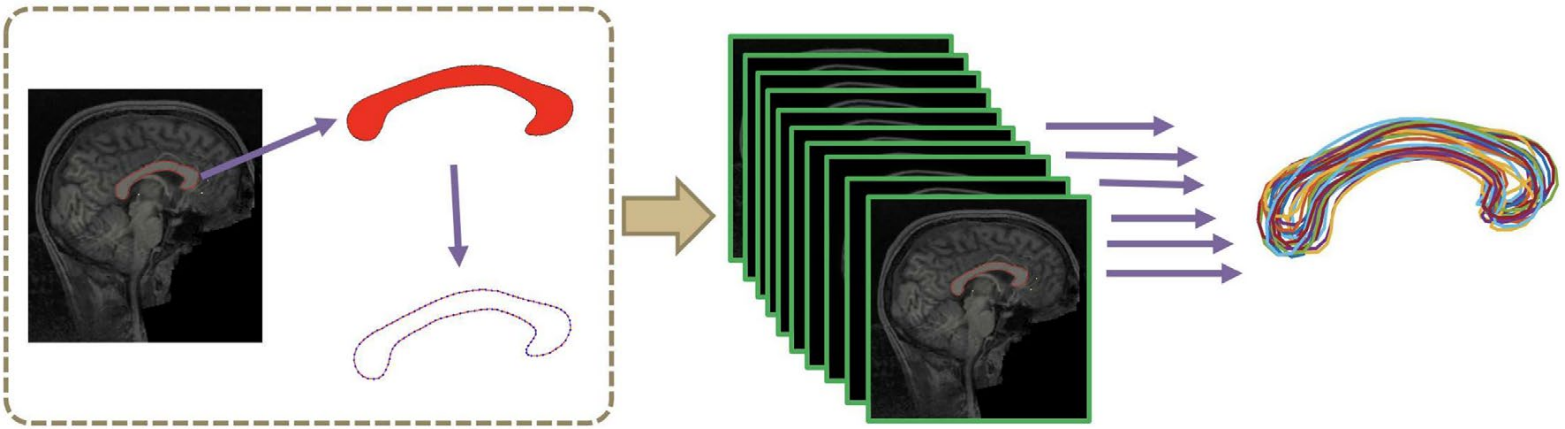
The processing steps for extracting the planar CC contour data.

In this data analysis, we considered the 818 subjects' genotype variables acquired by using the Human 610‐Quad BeadChip (Illumina Inc., San Diego, CA) in the ADNI‐1 database, which includes 620,901 SNPs. To reduce the population stratification effect, we used data from 749 Caucasians among all 818 subjects with complete imaging measurements at baseline. Quality control procedures included (i) call rate check per subject and per SNP marker, (ii) gender check, (iii) sibling pair identification, (iv) the Hardy–Weinberg equilibrium test, (v) marker removal by MAF, and (vi) population stratification. The second line preprocessing steps included removal of SNPs with (i) more than 5% missing values, (ii) MAF smaller than 5%, and (iii) Hardy–Weinberg equilibrium $p‐\text{value}<10^{-6}$. Remaining missing genotype variables were imputed as the modal value (see Huang et al. (2017) for the detailed procedures). After the quality control procedures, 500,216 SNPs for 700 subjects, including both healthy controls and individuals with AD or MCI (156 AD, 346 MCI, and 198 healthy controls), remained in the final data analysis. The detailed demographic information of these 700 subjects is shown in Table 1. For the 700 subjects, we also included their standard T1‐weighted MRI scans, which were performed on a variety of 1.5 Tesla MRI scanners with protocols individualized for each scanner, using volumetric 3‐dimensional sagittal MPRAGE or equivalent protocols with varying resolutions. To obtain the representation of CC, we used *FreeSurfer* (Fischl 2012) to process each MRI scan, including motion correction, non‐parametric non‐uniform intensity normalization, affine transform to the MNI305 atlas, intensity normalization, skull stripping, and automatic subcortical segmentation. Some quality control procedures were done on each output image data. Then, through the package *CCSeg* (Vachet et al. 2012), each T1‐weighted MRI image and tissue segmentation results were used to extract the planar CC contour data on the midsagittal slice, which contains 100 landmarks (see Figure 2).

**TABLE 1:** Demographic information of 700 subjects from ADNI‐1 study.

Diagnostic status	AD	LMCI	NC	Total
Sample size	156	346	198	700
Gender (F/M)	72/84	123/223	90/108	285/415
Age range (years)	[55.1, 90.9]	[54.4, 89.3]	[62, 89.6]	[54.5, 90.9]
Handedness (R/L)	146/10	315/31	184/14	645/55
APOE4 (0/1/2)	52/73/31	154/149/43	145/49/4	351/271/78

## 3 Methods

In this paper, we would like to identify the underlying causal pathways from genetic exposures to the neurocognitive outcome implicitly through the CC shape mediator. To achieve this goal, our proposed imaging genetics mediation analysis framework, S‐GMAS, includes three main components: (i) a fast genome‐wide association analysis, (ii) elastic CC shape representations, and (iii) a functional mediation analysis model with shape mediators. Specifically, a fast genome‐wide association analysis procedure (van Rossum and Kruijer 2023) is conducted to detect potential genetic exposures (e.g., causal variants) with significant genetic effects on the clinical outcome. Then the shape information is extracted from the 2D CC contour and represented as functions in linear Hilbert space. By treating these shape representations as functional phenotypes, a functional mediation analysis model is established to identify the underlying causal pathways from the genetic exposure to the neurocognitive outcome implicitly through the subcortical shapes. The workflow of our S‐GMAS is summarized in Figure 1.

Before detailing procedures in the three components, some notations are introduced here. Suppose we observe the subcortical brain structures, genetic variants, and clinical information for n unrelated subjects. Let g be a locus in the set of $N_{G}$ genetic markers, denoted as $G=\left\{g_{1},\ldots ,g_{N_{G}}\right\}$. For the *i*th subject, we denote that (i) $L_{i}$ is a m×2 matrix with m landmarks representing the coordinate information along the 2D CC contour at the middle‐sagittal slice; (ii) $x_{i}^{\left(g\right)}\in \left\{0,1\right\}$ is the observation associated with the genetic marker at the locus g (e.g., single‐nucleotide polymorphisms (SNPs)); (iii) $c_{i}\in {\mathbb{R}}^{p}$ is a p×1 vector including clinical confounders (e.g., gender, age, or handedness) along with the intercept; and (iv) $y_{i}\in \mathbb{R}$ is the neurocognitive outcome (e.g., Alzheimer's Disease Assessment Scale Cognitive Subscale 13 score [ADAS‐13]).

### 3.1 fastGWAS

We first consider the fastGWAS to discover significant genetic variants, that is, SNPs, influencing the clinical outcome. Specifically, for the genetic marker at locus g, we consider a linear mixed model as follows:

(1) $$y_{i}=x_{i}^{\left(g\right)}\nu^{\left(g\right)}+c_{i}^{⊤}u^{\left(g\right)}+\varepsilon_{i},i=1,\ldots ,n$$

where $x_{i}^{\left(g\right)}$ is the genetic variant at locus g, $\nu^{\left(g\right)}$ is the corresponding genetic effect on the neurocognitive outcome, and $u^{\left(g\right)}$ is the effect of confounders $c_{i}$ on the neurocognitive outcome. Conditional on a set of observed confounders $c_{i}$ and applying the FDR‐based correction of multiple testing, we can select a subset of SNPs $G_{0}$ including $N_{G_{0}}$ top SNPs with significant genetic effects on the neurocognitive outcomes. Detailed procedures about fastGWAS can be found in van Rossum and Kruijer (2023).

### 3.2 Elastic CC Shape Representation

Given the landmarks $L_{i}$ along the 2D CC contour at the middle‐sagittal slice, we first derive the normalized coordinate functions, $f_{i}\left(s\right)≐{\left(f_{i,1}\left(s\right),f_{i,2}\left(s\right)\right)}^{⊤}$, with $f_{i,j}\left(s\right):\left[0,1\right]\to \mathbb{R},j=1,2,$ in the *x*‐axis and the *y*‐axis, respectively, via removing two shape‐preserving transformations, that is, translation and scale, from the landmarks (Srivastava and Klassen 2016; Bookstein 1991). After that, we derive the square‐root velocity function (SRVF) representations (Srivastava et al. 2011), $q_{i}\left(s\right)≐{\left(q_{i,1}\left(s\right),q_{i,2}\left(s\right)\right)}^{⊤}$, for the *i*th subject, where $q_{i,j}\left(s\right):\left[0,1\right]\to \mathbb{R}$, $q_{i,j}\left(s\right)={\dot{f}}_{i,j}\left(s\right)/\sqrt{|{\dot{f}}_{i,j}\left(s\right)|}$, j=1,2. In particular, we can further determine the optimal rigid transformation (i.e., the rotation group action) and the non‐rigid registration (i.e., the re‐parameterization group action) for each SRVF representation through the following optimization problem:

(2) $$O_{i}^{*},\tau_{i}^{*}\left(s\right)={\text{arginf}}_{O\in \mathrm{SO}\;\left(2\right),\tau \left(s\right)\in \Gamma }∥q_{\mu }\left(s\right)-O\left(q_{i}\circ \tau \left(s\right)\right)\sqrt{\dot{\tau }\left(s\right)}∥$$

where $q_{\mu }\left(s\right)$ is the atlas shape, for example, the Karcher mean of ${\left\{q_{i}\left(s\right)\right\}}_{i=1}^{n}$, $O\in \mathrm{SO}\left(2\right)$ represents the rigid transformation with $\mathrm{SO}\left(2\right)$ the set of all 2×2 rotation matrices, that is, $\mathrm{SO}\left(2\right)=\left\{O\in {\mathbb{R}}^{2\times 2}|{\mathrm{OO}}^{⊤}=I_{2}\right\}$, and $\tau \left(s\right)$ represents the non‐rigid registration with Γ the set of all possible diffeomorphisms of $\left[0,1\right]$ that preserve the boundaries, that is, $\Gamma =\left\{\tau \left(s\right):\left[0,1\right]\to \left[0,1\right]|\tau \left(0\right)=0,\tau \left(1\right)=1\right\}$. To solve the optimization problem in (2), we consider an iterative approach. Specifically, given the warping function $\tau_{i}\left(s\right)$, the rotation matrix $O_{i}$ can be updated via the Procrustes analysis, while, given the updated rotation matrix, the optimal warping function can be determined via the dynamic programming algorithm (Srivastava and Klassen 2016). Then, we can obtain the aligned SRVF shape representations as follows:

(3) $$m_{i}\left(s\right)=O_{i}^{*}\left(q_{i}\circ \tau_{i}^{*}\left(s\right)\right)\sqrt{{\dot{\tau }}_{i}^{*}\left(s\right)},s\in \left[0,1\right],i=1,\ldots ,n$$

### 3.3 Functional Mediation Analysis With Shape Mediators

**FIGURE 3:**
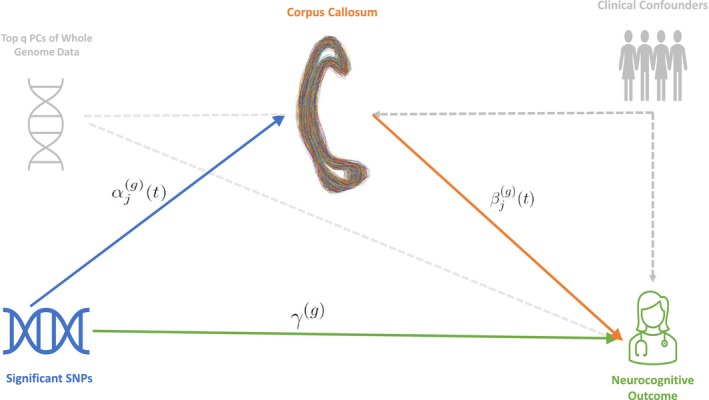
Path diagram of functional mediation analysis with CC shape mediators, including (i) a shape‐on‐scalar regression model and (ii) a scalar‐on‐shape regression model, to investigate the genetic causal effects on the neurocognitive outcome through the CC shape mediators.

To further investigate the genetic effect on the neurocognitive outcome mediated through the 2D CC shapes, we propose a mediation analysis framework using the structural equation modeling strategy and functional data analysis tools illustrated in Figure 3. Specifically, the proposed framework consists of a shape‐on‐scalar regression model (4) and a scalar‐on‐shape regression model (5) as follows:

(4) $$m_{i,j}\left(s\right)=x_{i}^{\left(g\right)}\alpha_{j}^{\left(g\right)}\left(s\right)+c_{i}^{⊤}\;\xi_{j}^{\left(g\right)}\left(s\right)+\eta_{i,j}^{\left(g\right)}\left(s\right)+\varepsilon_{i,j}\left(s\right),j=1,2$$

(5) $$y_{i}=x_{i}^{\left(g\right)}\gamma^{\left(g\right)}+\sum_{j=1}^{2}\;\int_{0}^{1}\;m_{i,j}\left(s\right)\beta_{j}^{\left(g\right)}\left(s\right)\mathrm{ds}+c_{i}^{Τ}\;\kappa^{\left(g\right)}+\delta_{i},i=1,\ldots ,n$$

where $x_{i}^{\left(g\right)}$ is the genetic exposure, that is, the significant SNP at locus g from the detected set $C_{0}$, and $c_{i}$ represents the clinical confounders. The coefficient functions $\alpha_{j}^{\left(g\right)}\left(s\right)$ and $\xi_{j}^{\left(g\right)}\left(s\right)$ respectively represent the genetic effect and effects caused by the clinical confounders. The individual functions $\eta_{i}^{\left(g\right)}\left(s\right)≐{\left(\eta_{i,1}^{\left(g\right)}\left(s\right),\ldots ,\eta_{i,d}^{\left(g\right)}\left(s\right)\right)}^{⊤}$, independent of $x_{i}^{\left(g\right)}$ and $c_{i}$, characterize the subject‐specific spatial variability and follows a Gaussian Process (GP) with mean 0 and covariance function $\Sigma_{\eta }^{\left(g\right)}\left(s,t\right)$. The measurement error $\varepsilon_{i,j}\left(s\right)$, independent of $\left\{x_{i}^{\left(g\right)},c_{i},\eta_{i}^{\left(g\right)}\left(s\right)\right\}$, follows a Gaussian distribution with mean 0 and variance $\sigma_{\varepsilon ,j}^{2}\left(s\right)$. In comparison, model (5) establishes the relationship among the neurocognitive outcome $y_{i}$, the genetic exposure $x_{i}^{\left(g\right)}$, and the shape mediator $m_{i}\left(s\right)$ while conditioning on the clinical confounders $c_{i}$. In particular, $\gamma^{\left(g\right)}$ represents the direct genetic effect of the genetic exposure, the coefficient functions ${\left\{\beta_{j}^{\left(g\right)}\left(s\right)\right\}}_{j=1}^{2}$ represent the effects from the shape mediators $m_{i}\left(s\right)$, and $\kappa^{\left(g\right)}$ represent the effects caused by the clinical confounders $c_{i}$. In addition, the errors ${\left\{\delta_{i}\right\}}_{i=1}^{n}$ are independent and Gaussian distributed with mean 0 and constant variance $\sigma_{\delta }^{2}$.

### 3.4 Causal Effect Estimands

Our aim is to quantify the causal effect of a genetic exposure (i.e., X) on the neurocognitive outcome (i.e., Y) mediated by the elastic shape mediator (i.e., M) given some clinical confounders (i.e., C). Using the potential outcome framework (Rubin 1978, 2005), we first formulate the causal estimands of interest, that is, the indirect effect and the direct effect. Let $M\left(s;x\right)$ denote the outcome of shape mediators under genetic exposure X=x and $Y\left(x,m\right)$ the potential outcome of the neurocognitive outcome when the genetic exposure and shape mediators are at the level x and $\left\{m\left(s\right),s\in D\right\}$ respectively. Given two levels of the genetic exposure $X_{i}=0$ and 1, the average direct effect (ADE), the average indirect effect (AIE), and the average total effect (ATE) can be derived as follows

(6) $$\mathrm{ADE}=\gamma ,\mathrm{AIE}=\sum_{j=1}^{d}\;\int_{0}^{1}\;\alpha_{j}\left(s\right)\beta_{j}\left(s\right)\mathrm{ds}$$

and ATE = ADE + AIE. In addition, we are also interested in the spatial AIE (SAIE), that is, the average indirect effect mediated through each $s\in \left[0,1\right]$, which is defined as

(7) $$\text{SAIE}\left(s\right)=\sum_{j=1}^{d}\;\alpha_{j}\left(s\right)\beta_{j}\left(s\right),s\in \left[0,1\right]$$

To interpret the estimated mediation effects causally, several assumptions are required. Specifically, we assume: (1) no interference between subjects (the stable unit treatment value assumption, SUTVA) (Rubin 1980); (2) no unmeasured confounders between the genetic exposure and the potential outcome given clinical confounders; (3) no unmeasured confounders between the shape mediator and the potential outcome given the genetic exposure and clinical confounders; (4) no unmeasured confounders between the genetic exposure and the mediator given clinical confounders; and (5) no confounders of the mediator‐outcome relationship that are themselves affected by the genetic exposure (VanderWeele 2015; Imai et al. 2010). A complete list of assumptions and their formal statements, along with detailed derivations of the causal estimands (ADE, AIE, ATE, and SAIE), is provided in Appendix A in Supporting Information.

### 3.5 Estimation and Inference on Causal Estimands

Given the proposed mediation framework, we can estimate all unknown parameters at locus $g\in G_{0}$, including (i) $\alpha_{j}^{\left(g\right)}\left(s\right),\xi_{j}^{\left(g\right)}\left(s\right),j=1,2$ in model (4), and (ii) $\gamma^{\left(g\right)},\beta_{j}^{\left(g\right)}\left(s\right),j=1,2,$ and $\kappa^{\left(g\right)}$ in model (5). The detailed estimation procedures can be found in Appendix B in Supporting Information. Then the naive plug‐in estimators for all the causal effects can be derived straightforwardly. Next, it is of great interest to conduct statistical inference to quantify the uncertainties of causal estimands, including the ADE, AIE, and SAIE defined in (6) and (7).

To achieve this goal, we use a wild bootstrap procedure (Lindquist 2012) to construct the confidence intervals for ADE and AIE, and the simultaneous confidence band (SCB) for the SAIE. Specifically, for a given confidence level ϑ, the bootstrap‐based 1−ϑ confidence interval of ADE and AIE, and the 1−ϑ SCB of SAIE at each locus $g\in G_{0}$ can be constructed respectively as follows:

(8) $$\left(\hat{\mathrm{ADE}}-C_{\mathrm{ADE}}\left(\vartheta \right),\hat{\mathrm{ADE}}+C_{\mathrm{ADE}}\left(\vartheta \right)\right)$$

(9) $$\left(\hat{\mathrm{AIE}}-C_{\mathrm{AIE}}\left(\vartheta \right),\hat{\mathrm{AIE}}+C_{\mathrm{AIE}}\left(\vartheta \right)\right)$$

(10) $$\left(\hat{\text{SAIE}}\left(s\right)-C_{\text{SAIE}}\left(\vartheta \right),\hat{\text{SAIE}}\left(s\right)+C_{\text{SAIE}}\left(\vartheta \right)\right)$$

where the definitions of $C_{\mathrm{ADE}}\left(\vartheta \right)$, $C_{\mathrm{AIE}}\left(\vartheta \right)$, and $C_{\text{SAIE}}\left(\vartheta \right)$ are provided along with the detailed bootstrap procedures in Appendix B in Supporting Information.

### 3.6 Simulation Studies and Sensitivity Analysis

To evaluate the performance of the proposed S‐GMAS framework, we conducted simulation studies using synthetic data that reflect the genetic–shape–cognition pathways. Genetic exposures, clinical confounders, functional shape mediators, and cognitive outcomes were generated under controlled models. Estimation accuracy was assessed using mean integrated absolute error (MIAE), mean integrated squared error (MISE), mean absolute error (MAE), and mean squared error (MSE) for both functional coefficients and causal effect estimands (ADE and AIE). Results demonstrate that S‐GMAS effectively recovers functional coefficients and mediation effects, with estimation accuracy improving as sample size increases. Complete simulation details and results are provided in Appendix C in Supporting Information.

We also conducted sensitivity analyses to examine the robustness of causal effect estimates under potential unmeasured confounding. A hidden confounder influencing both the mediator and outcome was introduced, with its effect systematically varied. The method showed resilience to modest confounding but exhibited bias under stronger confounding influences. Figure S2 in Appendix C in Supporting Information illustrates how increasing unmeasured confounding leads to greater deviation of the estimated AIE from the true value, as reflected in widening and shifting confidence bands. Full sensitivity analysis results are included in Appendix C in Supporting Information.

## 4 Results

**FIGURE 4:**
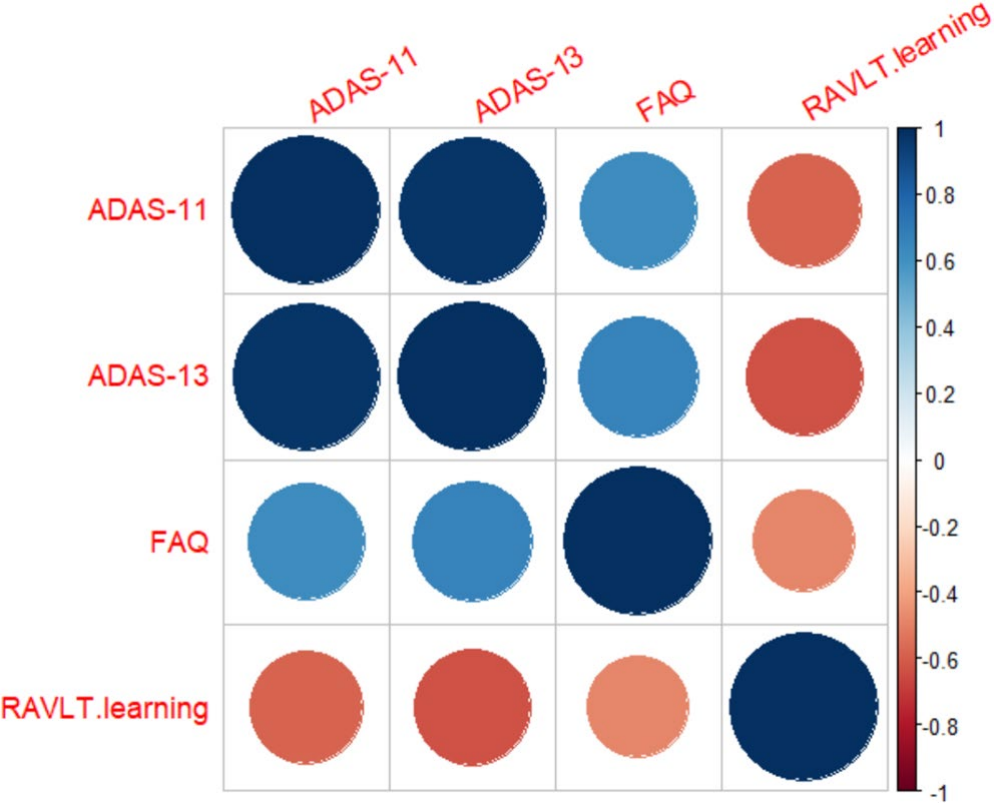
Correlations between the neurocognitive assessment scores.

The objective of this data analysis is to examine the genetic effect of each of 500,216 SNPs on the neurocognitive outcome mediated through the 2D CC phenotypes. For the neurocognitive outcome, we considered four different assessments including ADAS‐11, ADAS‐13, FAQ, and RAVLT.learning. Their full names and corresponding assessment items are listed in Table 2 (Kueper et al. 2018; Li et al. 2024). Figure 4 presents the correlations between these assessment scores, in which ADAS‐11, ADAS‐13, and FAQ are positively correlated, with higher values indicating more severe cognitive impairment, while RAVLT.learning is negatively correlated with them, with a lower value indicating more severe cognitive impairment. In addition, we also included several clinical confounders in our mediation analysis, such as age, gender, handedness, APOE4, and the top 5 PC scores of all the SNPs. In this data analysis, we considered the following two scenarios for the genetic exposure: (i) Polygenic Hazard Score (PHS) (Tan et al. 2017), where they used SNP effect sizes derived from the Cox Proportional Hazard Regression model; and (ii) the genome‐wide SNP data.

**TABLE 2:** Summary of neurocognitive assessments used in this study.

Score	Full name	Assessment
ADAS‐11	AD Assessment Scale‐Cognition 11 items	Memory, language, praxis domains
ADAS‐13	AD Assessment Scale‐Cognition 13 items	Memory, language, praxis domains, delayed word recall, orientation
FAQ	Functional Activities Questionnaire	Functional daily living impairment
RAVLT.learning	Learning score of the Rey Auditory Verbal Learning Test	Verbal learning and memory

**FIGURE 5:**
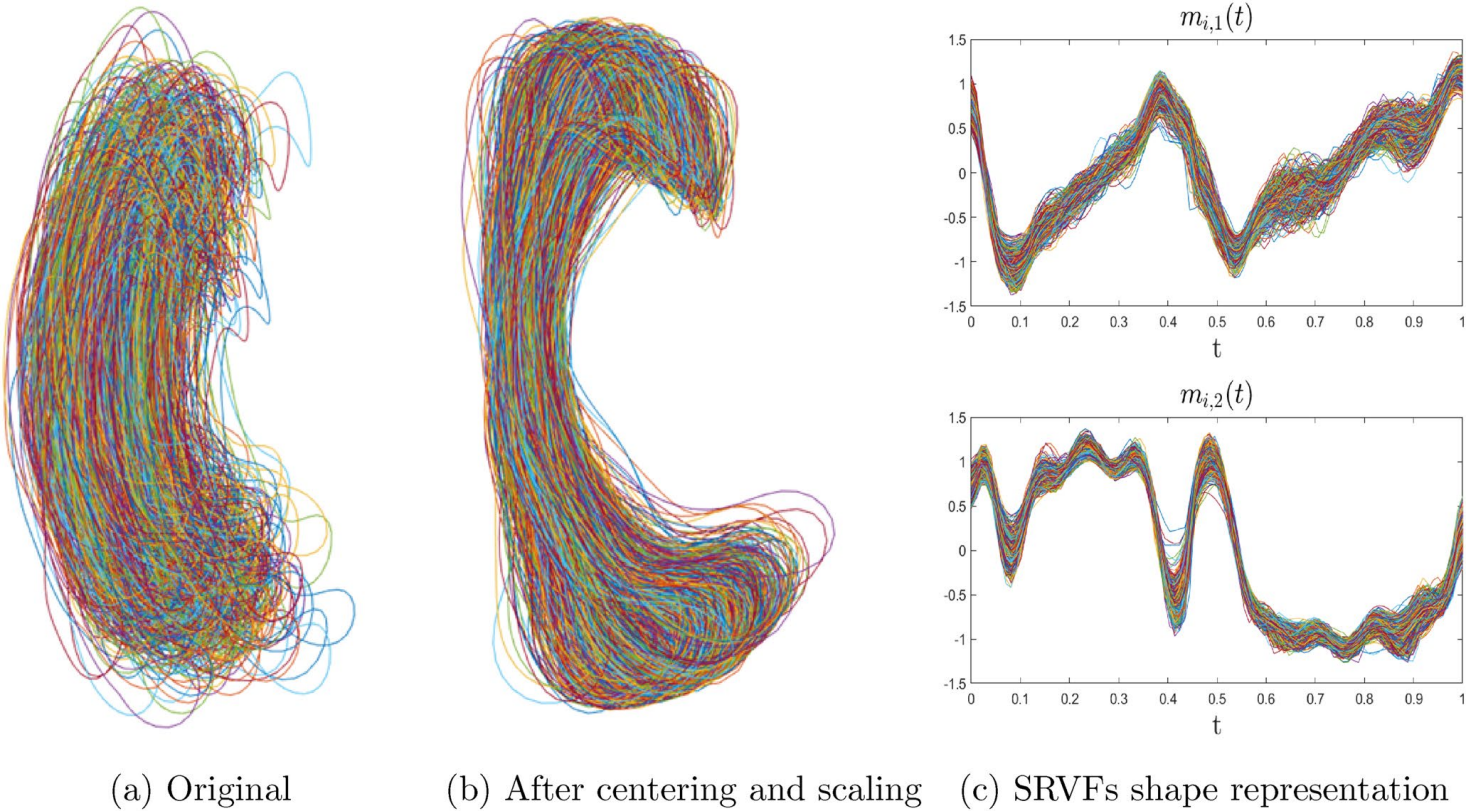
Visualization of CC shape data and corresponding representations: (a) landmarks sampled along the 2D contour of CC segmented at the middle‐sagittal slice from raw MRI brain images; (b) coordinate functions of landmarks with scaling and translation variabilities removed; (c) aligned SRVF shape representations.

As mentioned in Section 2, we extracted both the volumetric information of 5 CC subregions and the landmarks along the 2D CC contour at the middle‐sagittal slice. Therefore, in this data analysis, we considered two types of CC mediators, that is, multivariate mediators represented by CC subregion volumetric information and elastic shape mediators derived from the 2D CC landmarks. As an illustration in Figure 5, given the landmarks sampled along the 2D contour of CC segmented at the middle‐sagittal slice from raw MRI brain images (Figure 5a), we first removed the scaling and translation variabilities of the shape mediator (Figure 5b), then obtained the SRVF shape representations. The resulting aligned SRVFs $m_{i}\left(t\right)$ as shown in Figure 5c are then used for the following analysis.

### 4.1 Scenario 1: PHS

In this scenario, we first investigated whether PHS has significant genetic effects on each neurocognitive outcome, as defined in model (1). The estimated genetic effects ${\hat{\nu }}^{\left(\mathrm{PHS}\right)}$ for each clinical outcome and the corresponding *p*‐values are shown in Table 3. As expected, PHS aggregates the effect of genome‐wide SNPs and has a significant genetic effect on each neurocognitive outcome. In this scenario, we further investigated the genetic effects mediated by (i) the CC subregion volumetric information, and (ii) the elastic shape mediators as shown in Figure 5c.

**TABLE 3:** The estimated genetic effects of PHS and corresponding *p*‐values for each neurocognitive outcome.

Neurocognitive outcome	Genetic effect	*p*
ADAS‐11	2.9091	8.93E−07
ADAS‐13	4.3628	5.05E−07
FAQ	0.8539	2.54E−04
RAVLT.learning	−0.2774	5.52E−04

*Note:* The *p*‐values shown are unadjusted (raw) *p*‐values; Bonferroni correction was applied for multiple testing (significance threshold = 0.0125).

#### 4.1.1 CC Subregion Volumetric Mediators

To explore the genetic effect of PHS mediated by the CC subregion volumetric information on the neurocognitive outcomes, we directly applied Multiple Mediation Analysis (mma) (Yu and Li 2017) to calculate the AIE on each neurocognitive outcome. Based on the bootstrap resampling technique, we obtained the causal effects including ATE, ADE, AIE, and 95% confidence intervals of AIE jointly mediated by the total CC volumetric mediator as shown in Table 4. The estimated AIE and corresponding inference results of PHS on each clinical outcome mediated by each of the 5 CC subregion volumetric mediators are displayed in Table 5. However, given the 95% significant level, there is no statistically significant AIE mediated by either the total CC volume or each of the 5 CC subregion volumes.

**TABLE 4:** The causal effects of PHS on each clinical outcome jointly mediated by the CC subregion volumetric mediators.

Outcome	ATE	ADE	AIE	AIE 95% CI
ADAS‐11	2.9175	2.9175	−0.0000	[−0.0744, 0.0744]
ADAS‐13	4.3706	4.3661	0.0045	[−0.1138, 0.1229]
FAQ	0.9063	0.9094	−0.0030	[−0.0337, 0.0277]
RAVLT.learning	−0.3017	−0.2998	−0.0019	[−0.0120, 0.0082]

**TABLE 5:** AIE estimates and their inference results of PHS on each clinical outcome mediated by each of the 5 CC subregion volumes.

Subregion	ADAS‐11		ADAS‐13		FAQ		RAVLT.learning	
	AIE	AIE 95% CI	AIE	AIE 95% CI	AIE	AIE 95% CI	AIE	AIE 95% CI
Anterior	−0.0374	[−0.0978, 0.0230]	−0.0444	[−0.1392, 0.0505]	−0.0208	[−0.0498, 0.0082]	0.0045	[−0.0038, 0.0129]
Central	−0.0151	[−0.0762, 0.0461]	0.0173	[−0.0543, 0.0890]	−0.0031	[−0.0276, 0.0215]	−0.0044	[−0.0124, 0.0036]
MidAnterior	0.0311	[−0.0345, 0.0967]	−0.0330	[−0.1302, 0.0642]	−0.0102	[−0.0302, 0.0097]	0.0033	[−0.0042, 0.0107]
MidPosterior	0.0106	[−0.0365, 0.0578]	0.0332	[−0.0513, 0.1177]	0.0196	[−0.0060, 0.0453]	−0.0045	[−0.0143, 0.0054]
Posterior	0.0270	[−0.0190, 0.0730]	0.0592	[−0.0230, 0.1413]	0.0075	[−0.0096, 0.0246]	−0.0029	[−0.0091, 0.0034]

#### 4.1.2 Elastic Shape Mediators

Next, we investigated the genetic effects mediated by the elastic shape mediators utilizing our mediation framework proposed in Section 3.3, and the causal effects, including ATE, ADE, and AIE, along with their inference results are presented in Table 6, in which no significant AIE is detected in this scenario according to the 95% confidence interval.

**TABLE 6:** The causal effects of PHS on each clinical outcome mediated by the shape mediators.

Outcome	ATE	ADE	AIE	AIE 95% CI
ADAS‐11	2.9386	2.5753	0.3633	[−0.1284, 0.5571]
ADAS‐13	4.4159	3.7974	0.6185	[−0.2614, 0.9029]
FAQ	0.8751	0.6950	0.1801	[−0.0267, 0.2810]
RAVLT.learning	−0.2788	−0.2710	−0.0079	[−0.0243, 0.0069]

### 4.2 Scenario 2: Genome‐Wide SNPs

In this scenario, as described in Section 3.1, we first conducted the fastGWAS on each neurocognitive outcome to select a set of SNPs with significant genetic effects after the FDR‐based correction of multiple testing. The number of candidate SNPs detected on each neurocognitive outcome after fastGWAS, denoted as $N_{G_{0}}$, is displayed in Table 7. To further explore the causal pathways from the genetic exposures to each neurocognitive outcome through the CC subcortical structure, we first used the 5 CC subregion volumes as the multivariate mediators and conducted the multiple mediation analysis. However, after testing the genetic effect of each selected top SNP on the volumetric mediators and multiple testing adjustments, there is no significant SNP left after this step, which means that there is no causal pathway detected from the genetic markers to the neurocognitive outcome through the CC subregion volumetric representation.

**TABLE 7:** The number of detected SNPs in fastGWAS, $N_{G_{0}}$, and those with significant AIE, $N_{G_{c}}$, for each neurocognitive outcome.

Outcome	ADAS‐11	ADAS‐13	FAQ	RAVLT.learning
$N_{G_{0}}$	690	683	848	683
$N_{G_{c}}$	4	1	4	1

Next, we considered using the elastic CC shape representations to conduct the mediation analysis as illustrated in Section 3.3 for each selected candidate SNP $g\in G_{0}$ and neurocognitive outcome. We performed the estimation and inference procedures on the causal estimands as described in Section 3.5, then a subset of significant causal SNPs $G_{c}$ can be detected according to the confidence intervals of the SNPs. Table 7 displays the summary of the number of detected causal SNPs with significant AIE, denoted as $N_{G_{c}}$, for the clinical outcomes ADAS‐11, ADAS‐13, FAQ and RAVLT.learning respectively. The detailed information of the causal SNPs with significant AIE for each clinical outcome is shown in Table 8.

**TABLE 8:** The results of fastGWAS after *p*‐value adjustment for the detected causal SNPs, including the SNP name, chromosome (Chr), location (Pos), allele frequency (AllFreq), adjusted *p*‐value (Adj‐*p*Value), the genetic effect ${\hat{\nu }}^{\left(g\right)}$, along with its standard error in the parenthesis.

Outcome	SNP	Chr	Pos	AllFreq	Adj‐*p*Value	Effect
ADAS‐11	rs1556188	9	5486106	0.5686	3.6183E−04	−1.1516 (0.3240)
ADAS‐11	rs659561	10	84303712	0.9165	2.0217E−03	1.8284 (0.5937)
ADAS‐11	rs648958	10	84325187	0.0835	1.0425E−03	−1.9191 (0.5869)
ADAS‐11	rs659554	10	84351105	0.0545	3.8503E−02	−1.4562 (0.7040)
ADAS‐13	rs1556188	9	5486106	0.5686	3.5014E−04	−1.6906 (0.4746)
FAQ	rs1948397	14	86114130	0.8688	8.8440E−04	−0.6288 (0.1897)
FAQ	rs4646797	17	19560368	0.7631	2.9156E−04	0.5287 (0.1465)
FAQ	rs11657205	17	19623023	0.6688	6.2440E−04	0.4418 (0.1296)
FAQ	rs1989379	17	19631420	0.6206	1.2983E−02	0.3123 (0.1258)
RAVLT.learning	rs2343121	1	242700803	0.2575	1.8079E−02	0.1138 (0.0482)

The causal estimands of the detected causal SNPs $g\in G_{c}$ defined in Section 3.4, including the ATE, ADE, and AIE, the 95% confidence interval of the AIE, the mediation proportion (MP), and the corresponding gene, are summarized in Table 9. The MP, defined as the ratio of AIE to ATE, quantifies the proportion of the total genetic effect on neurocognitive outcomes that is mediated through shape alterations in the CC (Lee et al. 2024). It provides an interpretable summary of the relative importance of the indirect, shape‐mediated pathway. Across the reported SNP–outcome pairs with significant AIE, MP values range from approximately 13% to 37%, suggesting that shape‐based brain alterations mediate a meaningful portion of the genetic influence on neurocognitive outcomes. Notably, SNP rs659554 (nearest gene: NRG3) exhibits the highest MP (36.67%), indicating that over one‐third of its effect on ADAS‐11 is mediated through CC shape. In most cases, the ADE, AIE, and ATE estimates share the same directions, which means that if a SNP has a positive total/direct effect on the neurocognitive outcome, the indirect effect on the outcome implicitly through the mediators is also positive, and vice versa.

**TABLE 9:** The causal estimands and the corresponding genes for the detected causal SNPs.

Outcome	SNP	ATE	ADE	AIE	AIE 95% CI	MP (%)	Gene
ADAS‐11	rs1556188	−1.1703	−0.9753	−0.1950	[−0.3512, −0.0577]	16.66	PDCD1LG2
ADAS‐11	rs659561	1.8604	1.4085	0.4519	[0.1854, 0.7807]	24.29	NRG3
ADAS‐11	rs648958	−1.9579	−1.5509	−0.4070	[−0.7349, −0.1383]	20.79	NRG3
ADAS‐11	rs659554	−1.4770	−0.9354	−0.5416	[−0.9676, −0.1801]	36.67	NRG3
ADAS‐13	rs1556188	−1.7257	−1.3998	−0.3259	[−0.5863, −0.1128]	18.89	PDCD1LG2
FAQ	rs1948397	−0.6248	−0.5014	−0.1234	[−0.2274, −0.0312]	19.75	FLRT2
FAQ	rs4646797	0.5318	0.4261	0.1057	[0.0307, 0.1900]	19.88	ALDH3A2
FAQ	rs11657205	0.4423	0.3725	0.0698	[0.0024, 0.1389]	15.78	SLC47A2
FAQ	rs1989379	0.3202	0.2367	0.0835	[0.0186, 0.1547]	26.08	SLC47A2
RAVLT.learning	rs2343121	0.1108	0.1251	−0.0143	[−0.0274, −0.0032]	12.91	PLD5

Specifically, we identified four SNPs with significant shape‐mediated effects on ADAS‐11, corresponding to two genes: PDCD1LG2 and NRG3. Prior studies have linked PDCD1LG2 to AD risk and age of onset (Herold et al. 2016), while NRG3 has been associated with both AD (Herold et al. 2016; Lee et al. 2022) and other neurodegenerative conditions (Tian et al. 2021). Furthermore, SNP rs1556188 (PDCD1LG2) was also found to exert a significant indirect effect on ADAS‐13, consistent with its influence on ADAS‐11. In addition, four causal SNPs with significant shape‐mediated effects on FAQ were identified, corresponding to the genes FLRT2, ALDH3A2, and SLC47A2. Among these, ALDH3A2 has been implicated in AD through transcriptomic studies (Liang et al. 2008) and neurodegeneration (Rajeshwari et al. 2021), while FLRT2 has been linked to AD in phenome‐wide association analyses (Gouveia et al. 2022). Although SLC47A2 has not been directly associated with AD, it has been identified as a genetic locus influencing general cognitive ability (Davies et al. 2018). Lastly, for the neurocognitive outcome RAVLT.learning, the detected causal SNP corresponds to the gene PLD5, which has been associated with incident dementia in genome‐wide analyses (Harper et al. 2022), potentially related to incident AD.

**FIGURE 6:**
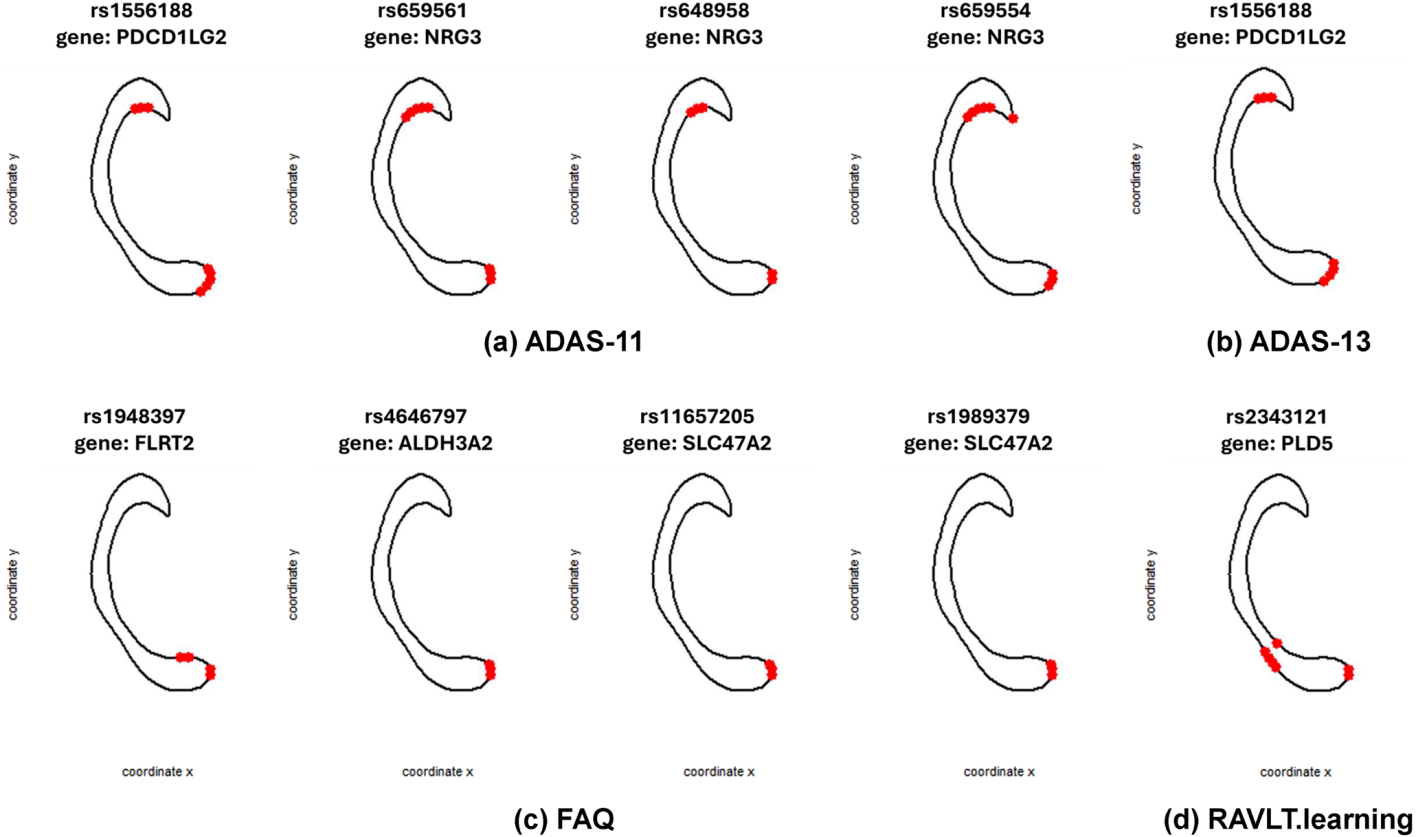
The CC subregions with significant SAIE for each detected causal SNP and each neurocognitive outcome respectively.

Besides the AIE, we also conducted statistical inferences on the SAIE to further investigate the spatial pattern of the mediation effect. For each detected causal SNPs and each neurocognitive outcome, the subregions with significant SAIE are shown in Figure 6. As observed from panels (a) and (b) of Figure 6, the effects of those genetic exposures on ADAS‐11 and ADAS‐13 are primarily mediated through two distinct subregions of the CC, located within the anterior and posterior regions. In comparison, for both FAQ and RAVLT.learning, there is only one subregion, within the posterior of CC, detected with significant SAIE, as shown in Figure 6c,d. It is worth noting that ADAS‐11 was originally developed for assessing dementia exhibiting severe cognitive impairments (Rosen et al. 1984), while ADAS‐13 is a modification of ADAS‐11, which includes all ADAS‐11 items and assesses two more tasks than ADAS‐11, that is, a test of delayed word recall and a number cancelation or maze task (Mohs et al. 1997; Kueper et al. 2018). It is consistent with the results that similar causal genes and spatial causal effect patterns were revealed by ADAS‐11 and ADAS‐13. Moreover, the FAQ was developed to distinguish between normal aging and mild senile dementia (Pfeffer et al. 1982), which was shown to be sensitive and responsive to dementia and disease progression in MCI (Mathuranath et al. 2005), and the RAVLT (Rey 1983) has been proven to be capable of efficiently identifying patients at both high and low risk for cognitive decline and subsequent dementia (Andersson et al. 2006). Thus the FAQ and RAVLT are usually used as accurate and reliable scales for early AD detection, while ADAS‐11 and ADAS‐13 are better assessed on patients with severe cognitive impairments or AD on‐site. It is aligned with the findings of only one subregion with significant SAIE detected for FAQ and RAVLT and an extra subregion detected for ADAS‐11 and ADAS‐13 with the disease progression and cognition impairment. Above all, it shows that different spatial causal effect patterns can be detected for different neurocognitive outcomes.

To visualize the relationship between shape variations and cognitive outcomes, we present representative CC shapes relative to the mean CC shape (middle panel) for ADAS‐11 in Figure S3 of Appendix D in Supporting Information. The CC shape in the left panel corresponds to a lower shape‐derived ADAS‐11 score, indicating better cognitive performance, while the shape in the right panel corresponds to a higher score, indicating poorer performance. These visualizations illustrate how specific morphological deformations of the CC are associated with differences in cognitive function.

These findings demonstrate that our proposed shape‐based mediation analysis can successfully detect causal genetic exposures with significant genetic effects on the clinical outcome directed through the shape representation of brain microstructure.

## 5 Discussion and Conclusion

In this study, we propose a genome‐wide mediation analysis framework that addresses high‐dimensional genetic exposures and shape mediators using SRVF‐based shape representations. The framework begins with a fast genome‐wide association analysis to identify significant genetic markers affecting shape mediators. We then implement a functional mediation approach, incorporating a shape‐on‐scalar model and a scalar‐on‐shape model, to explore the relationships between genetic markers, mediators, and neurocognitive outcomes. To estimate global and spatial mediation effects and conduct hypothesis testing, we employ a bootstrap resampling technique. Our analysis identifies distinct genetic‐to‐clinical outcome pathways mediated through the CC in AD, revealing varying spatial causal effect patterns for different clinical outcomes.

Although this paper offers a powerful tool for understanding genetic influences on neurocognitive outcomes, some limitations and future research directions remain. These are summarized below. First, while this work focuses on cross‐sectional mediation analysis using imaging and cognitive measures at a single time point, an important future extension is to adapt the framework for longitudinal data. Modeling repeated measures of subcortical shape and cognitive outcomes would allow for the investigation of dynamic mediation effects, enabling exploration of how morphological changes evolve over time and mediate the progression of genetic risk. Second, in the current implementation, mediation analyses are conducted for each candidate SNP individually, which enhances the interpretability of causal pathways. However, extending S‐GMAS to accommodate the joint mediation effects of multiple genetic variants is an important avenue for future research. Such an extension could provide a more comprehensive understanding of the collective genetic influence on neurocognitive outcomes via brain structural alterations. Third, observed confounders are addressed through direct adjustment in the shape‐on‐exposure and outcome‐on‐shape regression models. While this is effective under standard modeling assumptions, future work could further strengthen causal inference by incorporating methods such as propensity score modeling, inverse probability weighting, or doubly robust estimation. These approaches could improve robustness against residual confounding and enhance the causal interpretation of mediation effects. Fourth, although the current framework treats each neurocognitive assessment as a separate univariate outcome, a natural extension would be to jointly model these outcomes using a multivariate regression approach to account for potential correlations among cognitive traits. This remains an important methodological challenge and direction for future work. Finally, emerging evidence suggests that amyloid and tau pathology precede detectable structural brain changes in Alzheimer's disease. A valuable future extension of S‐GMAS would involve modeling tau and amyloid burden as earlier mediators or co‐mediators alongside structural alterations. Integrating PET‐based biomarkers into the mediation model could offer a more biologically informed representation of the progression from genetic risk to cognitive decline.

## Author Contributions

**Shengxian Ding:** conceptualization, methodology, data curation, formal analysis, writing – original draft and revision, visualization. **Rongjie Liu:** writing – review and editing. **Anuj Srivastava:** conceptualization, supervision, funding acquisition. **Richard S. Nowakowski:** supervision, writing – review and editing. **Li Shen:** writing – review and editing. **Paul M. Thompson:** data curation, writing – review and editing. **Heping Zhang:** writing – review and editing. **Chao Huang:** conceptualization, methodology, writing – review and editing, supervision, funding acquisition.

## Conflicts of Interest

The authors declare no conflicts of interest.

## Supporting information


**Data S1:** Supporting Information.

## Data Availability

Data used in the preparation of this article were obtained from the Alzheimer's Disease Neuroimaging Initiative (ADNI) database (adni.loni.usc.edu). The ADNI was launched in 2003 as a public‐private partnership, led by Principal Investigator Michael W. Weiner, MD. The primary goal of ADNI has been to test whether serial magnetic resonance imaging (MRI), positron emission tomography (PET), other biological markers, and clinical and neuropsychological assessment can be combined to measure the progression of mild cognitive impairment (MCI) and early Alzheimer's disease (AD). For up‐to‐date information, see www.adni‐info.org. The fastGWAS can be implemented by the R package “StatgenGWAS.” MATLAB code for deriving the elastic CC shape representations and finding the optimal transformation and registration, R code for conducting the proposed functional mediation framework with shape mediators can be found at https://github.com/Naomi‐Ding/S‐GMAS when the paper is accepted.
