# Supplementary material for: S‐GMAS: Genome‐Wide Mediation Analysis With Brain Subcortical Shape Mediators
Source: Hum Brain Mapp. 2025 Jul 31;46(11):e70297. doi: 10.1002/hbm.70297 (PMC12311987; doi:10.1002/hbm.70297)
Supplement: Supplementary file 1 — Data S1: Supporting Information. [file HBM-46-e70297-s001.pdf]

Web Appendix for “S-GMAS: Genome-wide mediation analysis with Brain Subcortical Shape Mediators”

Shengxian Ding,[1] Rongjie Liu,[2] Anuj Srivastava,[3] Richard S. Nowakowski,[4] Li Shen,[5] Paul M. Thompson,[6] Heping Zhang,[1] Chao Huang[7*]

[1]Department of Biostatistics, Yale University, New Haven, CT 06511, USA

[2]Department of Statistics, University of Georgia, Athens, GA 30602, USA

[3]Department of Statistics, Florida State University, Tallahassee, FL 32304, USA

[4]Department of Biomedical Sciences, Florida State University, Tallahassee, FL 32304, USA

[5]Department of Biostatistics, Epidemiology and Informatics, Perelman School of Medicine, University of Pennsylvania, Philadelphia, PA 19104, USA

[6]Imaging Genetics Center, USC Mark and Mary Stevens Neuroimaging and Informatics Institute, Keck School of Medicine, University of Southern California, Los Angeles, CA 90033, USA

[7]Department of Epidemiology & Biostatistics, University of Georgia, Athens, GA 30602, USA

[*]Correspondence: chaohuang@uga.edu

May 30, 2025

Web Appendix A. Model Assumption and Causal Identification

First, we introduce some potential outcome notations using capital letters to define the causal effects of interest. In particular, denote that $Y_i(x)$, corresponding to y_i, is the potential outcome under $X_i = x$, and $Y_i(x, \mathbf{m})$ is the potential outcome under $X_i = x$ and $\mathbf{M}_i = \mathbf{m}$. We also make the generally applied composition constraint by letting $Y_i(x) = Y_i(x, \mathbf{M}_i(x))$, where $\mathbf{M}_i(x) = \{\sum_{j=1}^{J} M_{i,j}(x,t),\ t \in [0,1]\}$ is the potential intermediate outcome under $X_i = x$ (Pearl et al., 2000). Given two arbitrary levels of the genetic exposure $X_i = x$ and x^*, the average direct effect

(ADE), the average indirect effect (AIE), and the average total effect (ATE) can be derived as follows

$$\begin{aligned}
\text{ADE} &= E[Y_i(x, \mathbf{M}_i(x^*)) - Y_i(x^*, \mathbf{M}_i(x^*))|\boldsymbol{c}_i] = \gamma^{(g)}(x - x^*), \\
\text{AIE} &= E[Y_i(x, \mathbf{M}_i(x)) - Y_i(x, \mathbf{M}_i(x^*))|\boldsymbol{c}_i] = (x - x^*)\sum_{j=1}^{d}\int_0^1 \alpha_j^{(g)}(t)\beta_j^{(g)}(t)dt. \\
\text{ATE} &= E[Y_i(x, \mathbf{M}_i(x)) - Y_i(x^*, \mathbf{M}_i(x^*))|\boldsymbol{c}_i] = \text{ADE} + \text{AIE},
\end{aligned}$$

In this paper, several assumptions are required to identify the causal effects, i.e., ADE, AIE, and ATE, from the observed data in our DMA. First, we make the stable unit treatment value assumption (SUTVA, Rubin (1980)): there is no interference between subjects, which implies that one individual's exposure assignment does not affect the outcome of others (details can be found in Imbens and Rubin (2015)). In addition, some no-unmeasured-confounding assumptions are also considered, which are extensions of the standard causal mediation assumptions in T. VanderWeele (2015):

Assumption I: $Y_i(x, \mathbf{m}) \perp\!\!\!\perp X_i | \boldsymbol{c}_i$, Assumption II: $Y_i(x, \mathbf{m}) \perp\!\!\!\perp \mathbf{M}_i(x) | \{X_i, \boldsymbol{c}_i\}$,

Assumption III: $\mathbf{M}_i(x) \perp\!\!\!\perp X_i | \boldsymbol{c}_i$, Assumption IV: $Y_i(x, \mathbf{m}) \perp\!\!\!\perp \mathbf{M}_i(x^*) | \boldsymbol{c}_i$.

where $A \perp\!\!\!\perp B | D$ represent that A is independent of B conditional on D. Assumption I implies that the levels of genetic exposure are independent of the potential outcome conditioning on the clinical confounders. Assumption II implies that the potential outcome is ignorable with respect to the distributional intermediate outcome conditioning on both the genetic exposure and clinical confounders. Assumption III states that the distributional intermediate outcome is ignorable with respect to the genetic exposure conditioning on the clinical confounders. Assumption IV implies that there is no mediator-outcome confounder that is affected by the exposure (T. J. VanderWeele & Vansteelandt, 2009).

Web Appendix B. Model Estimation & Inference on Causal Estimands

Model estimation

Recall that our proposed mediation framework consists of a shape-on-scalar regression model (1) and a scalar-on-shape regression model (2) as follows:

$$m_{i,j}(s) = x_i^{(g)}\alpha_j^{(g)}(s) + \boldsymbol{c}_i^\top \boldsymbol{\xi}_j^{(g)}(s) + \eta_{i,j}^{(g)}(s) + \epsilon_{i,j}(s),\ j = 1, 2, \tag{1}$$

$$y_i = x_i^{(g)}\gamma^{(g)} + \sum_{j=1}^{2}\int_0^1 m_{i,j}(s)\beta_j^{(g)}(s)ds + \boldsymbol{c}_i^\top \boldsymbol{\kappa}^{(g)} + \delta_i,\ i = 1, \ldots, n, \tag{2}$$

We can estimate all unknown parameters at locus $g \in \mathcal{G}_0$, including (i) $\alpha_j^{(g)}(s), \boldsymbol{\xi}_j^{(g)}(s),\ j = 1, 2$ in model (1), and (ii) $\gamma^{(g)}, \beta_j^{(g)}(s), j = 1, 2,$ and $\boldsymbol{\kappa}^{(g)}$ in model (2). First, model (1) can be viewed as a multivariate varying coefficient model (Zhu et al., 2012), the estimate of $\boldsymbol{\theta}_j^{(g)}(s)$ can be obtained via minimizing a weighted least squares function based on the multivariate local linear technique

$$\sum_{i=1}^{n}\sum_{k=1}^{n_v}\left\{m_{i,j}(s_k) - \boldsymbol{z}_i^\top\left(\boldsymbol{\theta}_j^{(g)}(s) + \dot{\boldsymbol{\theta}}_j^{(g)}(s_k)(s_k - s)\right)\right\}^2 K_{h_j}(s_k - s), \tag{3}$$

where $\boldsymbol{z}_i = \left(x_i^{(g)}, \boldsymbol{c}_i^\top\right)^\top$, $\boldsymbol{\theta}_j^{(g)}(s) = \left(\alpha_j^{(g)}(s), \left(\boldsymbol{\xi}_j^{(g)}(s)\right)^\top\right)^\top$, and $K_h(s) = h^{-1}K(s/h)$ is the scaled kernel function with a bandwidth h. Then we have

$$\hat{\boldsymbol{\theta}}_j^{(g)}(s) = \left(\boldsymbol{I}_{p+1} \otimes (1, 0)\right)\mathbf{U}(s, h_j)^{-1}\sum_{i=1}^{n}\sum_{k=1}^{n_v} K_{h_j}(s_k - s)\left(\boldsymbol{z}_i \otimes \boldsymbol{e}_{h_j}(s_k - s)\right)m_{i,j}(s), \tag{4}$$

where $\mathbf{U}(s, h_j) = \sum_{i=1}^{n}\sum_{k=1}^{n_v} K_{h_j}(s_k - s)\left(\boldsymbol{z}_i^{\otimes 2} \otimes \boldsymbol{e}_{h_j}(s_k - s)^{\otimes 2}\right)$, $\boldsymbol{e}_{h_j}(s_k - s) = (1, (s_k - s)/h_j)^\top$, $\boldsymbol{a}^{\otimes 2} = \boldsymbol{a}\boldsymbol{a}^\top$, $\otimes$ denotes the Kronecker product of two matrices, and $\boldsymbol{I}_p$ is an $p \times p$ identify matrix. Therefore, we can obtain the estimates of $\alpha_j^{(g)}(s)$ and $\boldsymbol{\xi}_j^{(g)}(s)$ given by $\left(1, \mathbf{0}_p^\top\right)\hat{\boldsymbol{\theta}}_j^{(g)}(s)$ and $\left(\mathbf{0}_p, \boldsymbol{I}_p\right)\hat{\boldsymbol{\theta}}_j^{(g)}(s)$ respectively, where $\mathbf{0}_p$ is a column vector of length p.

Subsequently, we approach model (2) by minimizing the following penalized least square function

$$\mathcal{L}_\lambda(\gamma^{(g)}, \beta_j^{(g)}(s), \boldsymbol{\kappa}^{(g)}) = \sum_{i=1}^{n}\left(y_i - x_i^{(g)}\gamma^{(g)} - \sum_{j=1}^{2}\int_0^1 m_{i,j}(s)\beta_j^{(g)}(s)ds - \boldsymbol{c}_i^\top\boldsymbol{\kappa}^{(g)}\right)^2 + \lambda\sum_{j=1}^{2}\left(\int_0^1 \ddot{\beta}_j^2(s)ds\right), \tag{5}$$

where λ is a constant smoothing parameter. First we express the functions $\beta_j^{(g)}(s)$ as a linear combination of L known basis functions $\phi_l(s)$, that is, $\beta_j^{(g)}(s) = \sum_{l=1}^{L} \phi_l(s) b_{l,j}^{(g)} = \mathbf{\Phi}(s)^\top \boldsymbol{b}_j^{(g)}$, where $\mathbf{\Phi}(s) = (\phi_1(s), \ldots, \phi_L(s))^\top$ and $\boldsymbol{b}_j^{(g)} = (b_{1,j}^{(g)}, \ldots, b_{L,j}^{(g)})^\top$. Under this expression, model (2) can be rewritten as follows

$$y_i = x_i^{(g)} \gamma^{(g)} + \sum_{j=1}^{2} \mathbf{\Lambda}_{i,j} \boldsymbol{b}_j^{(g)} + \boldsymbol{c}_i^\top \boldsymbol{\kappa}^{(g)} + \delta_i \tag{6}$$

where $\mathbf{\Lambda}_{i,j} = \int_0^1 m_{i,j}(s) \mathbf{\Phi}(s)^\top ds$. Then the solution of (5) is given by $\hat{\boldsymbol{\omega}}^{(g)} = (\mathbf{V}^\top \mathbf{V} + \lambda \mathbf{P})^{-1} \mathbf{V}^\top \boldsymbol{y}$, where $\mathbf{V} = \begin{pmatrix} x_1^{(g)} & \mathbf{\Lambda}_{1,1} & \mathbf{\Lambda}_{1,2} & \boldsymbol{c}_1^\top \\ \vdots & \vdots & \vdots & \vdots \\ x_n^{(g)} & \mathbf{\Lambda}_{n,1} & \mathbf{\Lambda}_{n,2} & \boldsymbol{c}_n^\top \end{pmatrix}$, $\boldsymbol{\omega}^{(g)} = \left(\gamma^{(g)}, (\boldsymbol{b}_1^{(g)})^\top, (\boldsymbol{b}_2^{(g)})^\top, (\boldsymbol{\kappa}^{(g)})^\top \right)^\top$, $\boldsymbol{y} = (y_1, \ldots, y_n)^\top$, and $\mathbf{P}$ is a matrix with element $P_{i,j}$ equal to zero if i or $j = 1$ and $\int_{\mathcal{S}} \ddot{\phi}_i^2(s) \ddot{\phi}_j^2(s) ds$ otherwise. Therefore, the estimates of the parameters of interest are given by $\hat{\gamma}^{(g)} = \left(1, \mathbf{0}_{2L+p}^\top\right) \hat{\boldsymbol{\omega}}^{(g)}$, $\hat{\beta}_1^{(g)}(s) = \mathbf{\Phi}(s)^\top \left(0, \mathbf{1}_L^\top, \mathbf{0}_{L+p}^\top\right) \hat{\boldsymbol{\omega}}^{(g)}$, $\hat{\beta}_2^{(g)}(s) = \mathbf{\Phi}(s)^\top \left(\mathbf{0}_{1+L}^\top, \mathbf{1}_L^\top, \mathbf{0}_p^\top\right) \hat{\boldsymbol{\omega}}^{(g)}$, and $\hat{\boldsymbol{\kappa}}^{(g)} = \left(\mathbf{0}_{1+2L}, \mathbf{1}_p\right) \hat{\boldsymbol{\omega}}^{(g)}$.

Estimation & inference on causal estimands

Given the estimated parameters in (1) and (2), the naive plug-in estimators for all the causal effects can be derived straightforwardly. Next, it is of great interest to perform inference on the direct effect (i.e., ADE) and indirect effects (i.e., AIE) through the shape mediators, and the SAIE.

Specifically, we use a wild bootstrap procedure to build the confidence intervals for ADE and AIE, and the simultaneous confidence band (SCB) for the SAIE. To produce a bootstrap sample $(x_i^{(g)}, y_i^*, \boldsymbol{m}_i(s)^*), i = 1, \ldots, n$, we implement the following data-generating process (DGP) (Lindquist, 2012) for $g \in \mathcal{G}_0$:

$$m_{i,j}^*(s) = x_i^{(g)} \hat{\alpha}_j^{(g)}(s) + \boldsymbol{c}_i^\top \hat{\boldsymbol{\xi}}_j^{(g)}(s) + v_i^* \hat{R}_{i,j}(s) \; s \in [0,1], \; j = 1, \ldots, d, \tag{7}$$

$$y_i^* = x_i^{(g)} \hat{\gamma}^{(g)} + \sum_{j=1}^{2} \int_0^1 m_{i,j}(s) \hat{\beta}_j^{(g)}(s) ds + \boldsymbol{c}_i^\top \hat{\boldsymbol{\kappa}}^{(g)} + v_i^* \hat{\delta}_i, \tag{8}$$

where $\hat{\alpha}_j^{(g)}(t), \hat{\boldsymbol{\xi}}_j(t), \hat{\beta}_j^{(g)}(t), \hat{\gamma}^{(g)}$ and $\hat{\boldsymbol{\kappa}}^{(g)}$ are estimates of the coefficient functions and scalar parameters in (1) and (2), while $\hat{R}_{i,j}(t) = m_{i,j}(t) - x_i^{(g)} \hat{\alpha}_j^{(g)}(s) - \boldsymbol{c}_i^\top \hat{\boldsymbol{\xi}}_j^{(g)}(s)$ and $\hat{\delta}_i$ are the ith residuals, and v_i^* are independent and identically distributed as $v_i^* = \begin{cases} 1 & \text{with probability } 0.5 \\ -1 & \text{with probability } 0.5 \end{cases}$.

Then for a given confidence level ϑ, the bootstrap procedure for constructing the $1-\vartheta$ confidence interval of ADE and AIE, and the $1-\vartheta$ SCB of SAIE at each locus $g \in \mathcal{G}_0$ is performed as follows:

1. Estimate the coefficient functions $\hat{\alpha}_j^{(g)}(s), \hat{\boldsymbol{\xi}}_j(s), \hat{\beta}_j^{(g)}(s), \hat{R}_{i,j}(s)$, and the scalar parameters $\hat{\gamma}^{(g)}, \hat{\boldsymbol{\kappa}}^{(g)}, \hat{\delta}_i$ in (1) and (2);

2. Estimate the ADE, AIE and SAIE based on the estimates in Step 1, denoted as $\widehat{ADE} = \hat{\gamma}^{(g)}, \widehat{AIE} = \sum_{j=1}^{2}\int_0^1 \hat{\alpha}_j^{(g)}(s)\hat{\beta}_j^{(g)}(s)ds$ and $\widehat{SAIE}(s) = \sum_{j=1}^{2} \hat{\alpha}_j^{(g)}(s)\hat{\beta}_j^{(g)}(s)$;

3. Independently generate a random sample $v_i^{(b)}$, then construct $\left(x_i, y_i^{(b)}, \boldsymbol{m}_i(t)^{(b)}\right), i = 1, \ldots, n$, using the wild bootstrap DGP described in (7) and (8);

4. Based on the resampled data, refit the models and get the estimated ADE, $\widehat{ADE}^{(b)}$, the estimated AIE, $\widehat{AIE}^{(b)}$, and the estimated SAIE, $\widehat{SAIE}(s)^{(b)}$ respectively;

5. Repeat Step 3 and 4 B times to obtain the sequences $\left\{\widehat{ADE}^{(b)}, \widehat{AIE}^{(b)}, \widehat{SAIE}(s)^{(b)}\right\}_{b=1}^{B}$;

6. The $1-\vartheta$ confidence interval of ADE and AIE can be derived as

$$\left(\widehat{ADE} - \mathcal{C}_{ADE}(\vartheta),\ \widehat{ADE} + \mathcal{C}_{ADE}(\vartheta)\right), \tag{9}$$

$$\left(\widehat{AIE} - \mathcal{C}_{AIE}(\vartheta),\ \widehat{AIE} + \mathcal{C}_{AIE}(\vartheta)\right), \tag{10}$$

   where $\mathcal{C}_{ADE}(\vartheta)$ and $\mathcal{C}_{AIE}(\vartheta)$ are the $1-\vartheta$ empirical percentile of $\left\{\widehat{ADE}^{(b)}, \widehat{AIE}^{(b)}\right\}_{b=1}^{B}$; the $1-\vartheta$ simultaneous confidence band of SAIE can be derived as

$$\left(\widehat{SAIE}(s) - \mathcal{C}_{SAIE}(\vartheta),\ \widehat{SAIE}(s) + \mathcal{C}_{SAIE}(\vartheta)\right), \tag{11}$$

   where $\mathcal{C}_{SAIE}(\vartheta)$ is the $1-\vartheta$ empirical percentile of $\{\sup_{s\in[0,1]} |\widehat{SAIE}(s)^{(b)}|\}_{b=1}^{B}$.

Web Appendix C. Simulation Studies

To examine the proposed framework, we generated synthetic curves to explore the estimation performance of the unknown functions and parameters in the models and investigate the causal estimands, i.e., the averaged direct effect (ADE) and the averaged indirect effect (AIE). As the first step fastGWAS has been validated in van Rossum and Kruijer, 2023, we only focus on the mediation framework. To be specific, we simulated the dataset from the following models:

$$m_{i,j}(t_k) = \mu_j(t_k) + x_i\alpha_j(t_k) + \boldsymbol{c}_i^\top \boldsymbol{\xi}_j(t_k) + \eta_{i,j}(t_k) + \epsilon_{i,j}(t_k),\ t_k \in [0,1],\ i = 1,\dots,n,\ j = 1,2,$$

$$y_i = \nu + x_i\gamma + \sum_{j=1}^{2} \int_0^1 m_{i,j}(t)\beta_j(t)dt + \boldsymbol{c}_i^\top \boldsymbol{\kappa} + \delta_i,\ i = 1,\dots,n,$$

where $t_1 = 0 \le t_2 \le \cdots \le t_{n_v} = 1$ with equal distance. We set the genetic exposure and clinical confounders $(x_i, \boldsymbol{c}_i) = (x_i, c_{i,1}, c_{i,2})^\top$, in which we independently simulated it from a multivariate normal distribution $N(\mathbf{0}_3, \boldsymbol{\Sigma})$ for $i = 1,\dots,n$, where $\boldsymbol{\Sigma}$ is a 3×3 matrix with elements $0.6^{|j'-j|}$ for $j, j' = 1,2,3$. The individual function $\eta_{i,j}(t)$ admits the Karhunen-Loeve expansion as $\eta_{i,j}(t) = \zeta_{i,j,1}\psi_{j,1}(t) + \zeta_{i,j,2}\psi_{j,2}(t)$ where $\zeta_{i,j,1} \sim N(0, \lambda_1 = 0.5), \zeta_{i,j,2} \sim N(0, \lambda_2 = 1)$, $\psi_{j,1}(t) = \sqrt{2}\sin(2\pi t)$ and $\psi_{j,2}(t) = \sqrt{2}\cos(2\pi t)$ for $j = 1,2$.

The functional coefficients $\alpha_j(t), \boldsymbol{\xi}_j(t), \beta_j(t)$ are generated based on the multiplication of randomly generated numbers and the B-spline basis functions with the number of knots 30 at order 6. The ground truth of these functions is displayed in Figure 1, while $\mu_1(t) = (t - 0.1)^2$ and $\mu_2(t) = t^3$, which is treated as Scenario 1. To explore the effect of different severity of the indirect effects, we set the functional coefficients in model 2 as $\boldsymbol{\beta}(t)/4$ and keep the others the same, which is Scenario 2. To clarify, in Scenario 1, AIE is around 7.3114, larger than ADE (=3), while in Scenario 2, AIE is around 1.8278, smaller than ADE. Furthermore, we set $\nu = 1, \gamma = 3$, and $\boldsymbol{\kappa} = (2,4)^\top$, and the random errors $(\epsilon_{i,1}(t_k), \epsilon_{i,2}(t_k) \sim N\left((0,0)^\top, diag\left(\sigma^2_{\epsilon,1}, \sigma^2_{\epsilon,2}\right)\right), \delta_i \sim N(0, \sigma^2_\delta)$, where $(\sigma^2_{\epsilon,1}, \sigma^2_{\epsilon,2}, \sigma^2_\delta) = (1^2, 0.5^2, 0.05^2)$.

We measured the estimation performance for the functional coefficients using the mean integrated absolute error (MIAE) and the mean integrated squared error (MISE) and for the parameters using the mean absolute error (MAE) and the mean squared error (MSE) based on 200 replications. We also compared the results for different sample sizes, $n = 200$ and $n = 500$, respectively, which are shown in Table 1 and Table 2.

Moreover, to investigate the causal estimands, we compared the estimated ADE and AIE with the

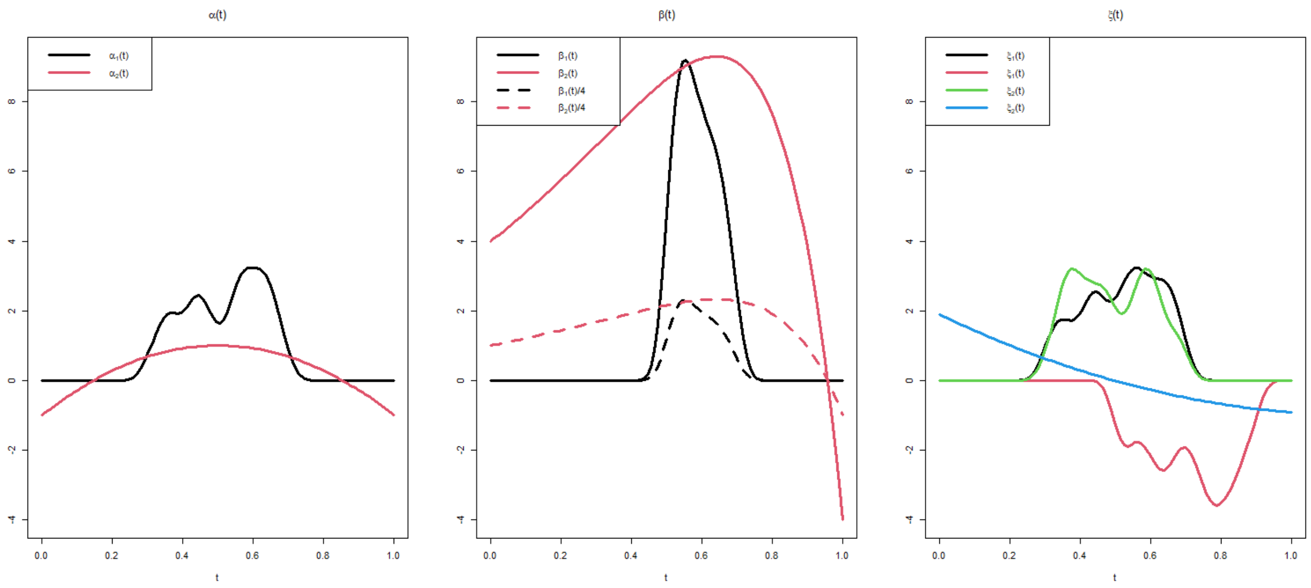

Figure 1: The functional coefficients $\alpha_j(t), \boldsymbol{\xi}_j(t)$ and $\beta_j(t)$ for $j = 1, 2$ generated by B-spline basis.

Table 1: Estimation errors of the functional coefficients $\alpha_j(t), \boldsymbol{\xi}_j(t), \beta_j(t), j = 1, 2$ and the parameters γ and $\boldsymbol{\kappa}$ in Scenario 1 based on 200 replications.

n	Functions	$\alpha_1(t)$	$\alpha_2(t)$	$\xi_{1,1}(t)$	$\xi_{1,2}(t)$	$\xi_{2,1}(t)$	$\xi_{2,2}(t)$	$\beta_1(t)$	$\beta_2(t)$	Parameters	γ	κ_1	κ_2
200	MIAE	0.1049	0.0955	0.0899	0.0873	0.0881	0.0808	0.1591	0.1451	MAE	0.0439	0.0706	0.0569
	MISE	0.0182	0.0143	0.0129	0.0125	0.0128	0.0107	0.0422	0.0370	MSE	0.0030	0.0080	0.0049
500	MIAE	0.0657	0.0592	0.0600	0.0566	0.0603	0.0536	0.1018	0.1182	MAE	0.0236	0.0392	0.0473
	MISE	0.0070	0.0057	0.0059	0.0052	0.0059	0.0045	0.0173	0.0366	MSE	0.0009	0.0025	0.0031

ground truth using the mean, bias, standard deviation, MAE and MSE based on 200 replications for both scenarios, as shown in Table 3.

Furthermore, we explored the coverage probability for the confidence bands of AIE obtained from the bootstrap method under different scenarios. Here we considered three scenarios: besides the two scenarios mentioned above, we consider another scenario as Scenario 3, in which we treat the first confounder as the genetic exposure, that is, we switch the original exposure x_i and one confounder $c_{i,1}$, correspondingly their coefficients $\alpha_j(t)$ and $\xi_{1,j}(t)$. The ground truth of AIE

Table 2: Estimation errors of the functional coefficients $\alpha_j(t), \boldsymbol{\xi}_j(t), \beta_j(t), j = 1, 2$ and the parameters γ and $\boldsymbol{\kappa}$ in Scenario 2 based on 200 replications.

n	Functions	$\alpha_1(t)$	$\alpha_2(t)$	$\xi_{1,1}(t)$	$\xi_{1,2}(t)$	$\xi_{2,1}(t)$	$\xi_{2,2}(t)$	$\beta_1(t)$	$\beta_2(t)$	Parameters	γ	κ_1	κ_2
200	MIAE	0.1119	0.0996	0.0958	0.0907	0.0943	0.0845	0.0854	0.0695	MAE	0.0270	0.0457	0.0275
	MISE	0.0206	0.0156	0.0147	0.0134	0.0146	0.0116	0.0122	0.0086	MSE	0.0011	0.0032	0.0012
500	MIAE	0.0701	0.0616	0.0637	0.0586	0.0640	0.0558	0.0566	0.0487	MAE	0.0152	0.0244	0.0204
	MISE	0.0079	0.0061	0.0066	0.0056	0.0066	0.0049	0.0053	0.0041	MSE	0.0004	0.0010	0.0006

Table 3: Mean performance of the averaged direct effect (ADE) and the averaged indirect effect (AIE) based on 200 replications.

	Scenario 1						Scenario 2					
	Our Method				MMA		Our Method				MMA	
	$n = 200$		$n = 500$		$n = 500$		$n = 200$		$n = 500$		$n = 500$	
	ADE	AIE	ADE	AIE	ADE	AIE	ADE	AIE	ADE	AIE	ADE	AIE
True	3.0000	7.3114	3.0000	7.3114	3.0000	7.3114	3.0000	1.8278	3.0000	1.8278	3.0000	1.8278
Mean	3.0048	7.2833	3.0031	7.2959	3.0005	7.6045	3.0043	1.8152	3.0037	1.8190	3.0009	1.9013
Bias	-0.0048	0.0281	-0.0031	0.0155	-0.0005	-0.2931	-0.0043	0.0127	-0.0037	0.0088	-0.0009	-0.0735
Standard deviation	0.0546	0.3102	0.0298	0.1860	0.2012	0.5839	0.0335	0.0832	0.0193	0.0494	0.1101	0.1570
MAE	0.0439	0.2556	0.0236	0.1506	0.1593	0.5378	0.0270	0.0682	0.0152	0.0399	0.0863	0.1430
MSE	0.0030	0.0965	0.0009	0.0347	0.0403	0.4251	0.0011	0.0070	0.0004	0.0025	0.0121	0.0299

Table 4: The coverage probability for the confidence bands of AIE based on 500 bootstraps at the significant level $\tau = 0.05$ and $\tau = 0.01$ respectively.

Settings	Scenario 1 $(AIE = 7.3114)$			Scenario 2 $(AIE = 1.8278)$			Scenario 3 $(AIE = -3.8753)$	
	Our Method		MMA	Our Method		MMA	Our Method	
n	200	500	500	200	500	200	500	500
$\tau = 0.05$	0.940	0.950	1.000	0.915	0.935	0.990	0.910	0.945
$\tau = 0.01$	0.985	0.990	1.000	0.980	0.975	0.995	0.965	0.990

for each scenario is present in Table 4, as well as the corresponding coverage probability for the confidence bands of AIE based on 500 bootstrap samples. Naturally, when the sample size n is larger, the coverage probability is closer to the nominal value $1 - \tau$.

Sensitivity Analysis

While calculating the causal estimands, we assume that there are no unobserved confounders. However, this assumption could be violated in reality, causing the error in causal identification. To investigate the influence of unobserved confounders, we simulated a dataset similar to the previous setting while adding one more variable w_i as the unobserved confounder. To be specific, the true model should be the following:

$$m_{i,j}(t) = \mu_j(t) + x_i\alpha_j(t) + \boldsymbol{c}_i^\top \boldsymbol{\xi}_j(t) + w_i\vartheta_j(t) + \eta_{i,j}(t) + \epsilon_{i,j}(t),\ i = 1, \ldots, n,\ j = 1, 2,$$

$$y_i = \nu + x_i\gamma + \sum_{j=1}^{2} \int_0^1 m_{i,j}(t)\beta_j(t)dt + \boldsymbol{c}_i^\top \boldsymbol{\kappa} + w_i\iota + \delta_i,\ i = 1, \ldots, n,$$

where $\vartheta_j(t)$ represents the effect of the unobserved confounder on the mediators, while ι represents its effect on the clinical outcome. Here we set $\boldsymbol{\vartheta}(t) = (\vartheta_1(t), \vartheta_2(t)) = (r_1(3 - t^2), r_2 \cdot 2t)$, where r_1 and r_2 control the effect $\vartheta_1(t)$, ranging from 0 to 0.5 respectively. Moreover, we noticed

that the variation of ι does not change the estimates of $\beta_j(t), j = 1, 2$, neither does the average indirect effect (AIE), i.e., $\sum_{j=1}^{2} \alpha_j(s)\beta_j(s)$. Given this, we fixed $\iota = 1$ for all settings of (r_1, r_2).

The confidence bands of the average indirect effect (AIE) with different levels of effects caused by the unobserved confounder w_i with $n = 200, \iota = 0$ are shown in Figure 2. When $(r_1, r_2) = (0, 0)$, the true model does not include the unobserved variable w_i. It means our assumption is true, so the estimation and confidence bands for AIE should be valid, which is consistent with the first box in the figure. It's obvious that when $r_2 \leq 0.2$, the confidence bands cover the ground truth when r_1 is relatively small. For example, when $r_2 = 0.2$, the confidence bands do not cover the truth for any positive r_1; while $r_2 = 0.1$, the confidence bands deviate from the truth when $r_1 \geq 0.3$.

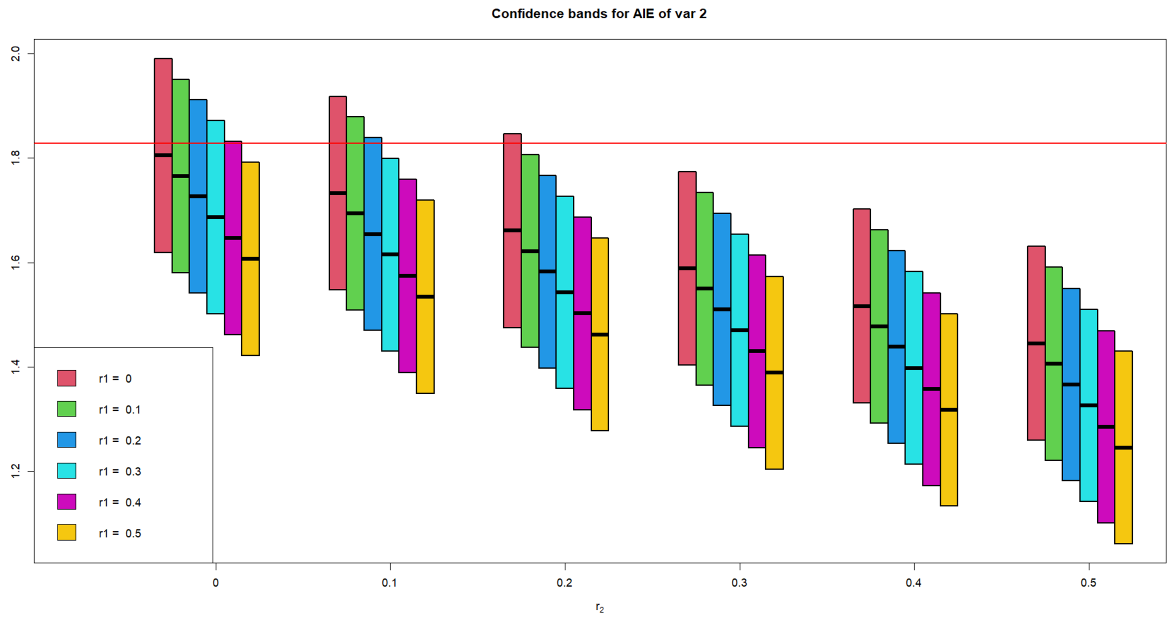

Figure 2: The confidence bands for the AIE for different levels of effects caused by the unobserved confounder w_i for $n = 200$. The x-axis denotes the value of r_2, and each colored box denotes different values of r_1. The red horizontal line represents the ground truth of AIE.

Web Appendix D. Additional Results in ADNI CC Data Analysis

To visualize the relationship between shape variations and cognitive outcomes, we present representative CC shapes relative to the mean CC shape (middle panel) for ADAS-11 in Figure 3. The CC shape in the left panel corresponds to a lower shape-derived ADAS-11 score, indicating better cognitive performance, while the shape in the right panel corresponds to a higher

score, indicating poorer performance. These visualizations illustrate how specific morphological deformations of the CC are associated with differences in cognitive function.

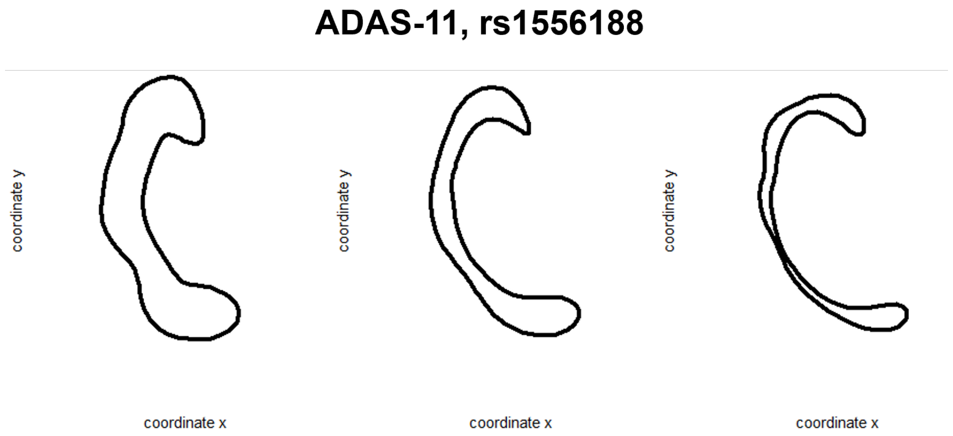

Figure 3: Representative CC shapes corresponding to a lower (left) and higher (right) values of the shape-derived ADAS-11 scores, compared with the mean CC shape (middle).

References

Imbens, G. W., & Rubin, D. B. (2015). *Causal inference in statistics, social, and biomedical sciences*. Cambridge University Press.

Lindquist, M. A. (2012). Functional causal mediation analysis with an application to brain connectivity. *Journal of the American Statistical Association*, *107*(500), 1297–1309.

Pearl, J., et al. (2000). Models, reasoning and inference. *Cambridge, UK: CambridgeUniversityPress*, *19*.

Rubin, D. B. (1980). Randomization analysis of experimental data: The fisher randomization test comment. *Journal of the American Statistical Association*, *75*(371), 591–593.

van Rossum, B.-J., & Kruijer, W. (2023). Statgengwas: Genome wide association studies. https://biometris.github.io/statgenGWAS/index.html

VanderWeele, T. (2015). *Explanation in causal inference: Methods for mediation and interaction*. Oxford University Press.

VanderWeele, T. J., & Vansteelandt, S. (2009). Conceptual issues concerning mediation, interventions and composition. *Statistics and its Interface*, *2*(4), 457–468.

Zhu, H., Li, R., & Kong, L. (2012). Multivariate varying coefficient model for functional responses. *Annals of statistics*, *40*, 2634–2666.
